# Neglected Opportunities—Agricultural Shows

**Published:** 1891-09

**Authors:** 


### NEGLECTED OPPORTUNITIES—AGRICULTURAL SHOWS.

Although the original *raison d'être* of Agricultural Shows is often lost sight of in the money-making schemes of local tradesmen and jockey-horse-owners, which corrupt the intentions of the founders, yet it is always acknowledged that the object of a show of animals is, by comparison of their relative merits, to determine which is the better animal, and by awarding such animal a badge of distinction, to attract attention to it, and thereby reward the intelligence of the owner, and advertise the animal for the benefit of the community. The award of prizes to an animal should indicate to the community that the animal receiving it is of an excellent and advantageous type, and should be a guarantee to the public, that such animal has no detectable defects, and that it is a safe, good animal to breed from, and that it is the standard towards which they should bring their own animals.

Unfortunately this is not always so. Competent judges are not always selected, even if competent on special subjects, the judges are often ignorant and narrow-minded, outside of the one class of animal they are interested in themselves. Judges are sometimes dishonest.

Does the veterinarian throughout the country take his proper position in regard to agricultural shows? We can safely assert, that he does not, and that he should. He, of all others, is one, who should be one of all Boards of Judges, to decide that a stallion or mare is deserving of a prize, that such animal has not a defective hock, an anchylozed ring-bone or a respiratory trouble, which, however handsome the animal may otherwise be, it will transmit to its progeny, and injure rather than benefit the community. He is the one to detect evidences of tuberculosis and other troubles which should render a bull or a cow unfit to receive a prize. He is the one to instruct the public that a thoroughbred, no matter how fashionable his pedigree, should not be awarded a blue ribbon if it has defects which are hereditary.

Now is the season of agricultural shows; how many veterinarians are judges at them? a very few; and to whom belongs the blame? It is too late now to remedy the matter this year; but, now is the time to remedy it for next year. Let every veterinarian visit his county fair, and examine the animals critically, note the errors of judging; which are due to want of proper anatomical and physiological knowledge, which could have been supplied by a veterinarian. Then write the county newspaper, a just review of the merits of the animals, and of the prizes awarded, leaving out all personalities and prejudice, and let time show the community, in the products of these animals that the expert was right.

THE JOURNAL will be glad to have these reviews and criticisms of the various agricultural fairs, to use for a general review of the results obtained from such fairs. The manner of conducting horse-racing at fairs is an important subject, as it may be of great benefit to the horse industry if properly managed, and may produce serious harm if improperly governed.

A horse show, a cattle show, a show of any industry should be an educator to the people, and the merits of the living articles should be explained by the veterinarian to those who are less instructed. If he goes no further than to make a careful study of the breeds and individual qualities of the animals, the veterinarian will be rewarded by the education he obtains himself.

